# Self-Reported Dietary Management Behaviors and Dietary Intake among Chinese Adults with Diabetes: A Population-Based Study

**DOI:** 10.3390/nu14235178

**Published:** 2022-12-05

**Authors:** Yixu Liu, Dongmei Yu, Jiayou Luo, Shuya Cai, Ping Ye, Zhenzhen Yao, Miyang Luo, Liyun Zhao

**Affiliations:** 1 Xiangya School of Public Health, Central South University, Changsha 410008, China; 2 Key Laboratory of Trace Element Nutrition of National Health Commission, Chinese Center for Disease Control and Prevention, National Institute for Nutrition and Health, Beijing 100050, China; 3 Yuxi Center for Disease Control and Prevention, Yuxi 653100, China

**Keywords:** diabetes, dietary management, diet quality, dietary intake, dietary adherence, dietary recommendations, China

## Abstract

Few studies have analyzed the implementation of dietary management in Chinese adults with diabetes. Thus, we assessed and compared dietary intake and diet quality between diabetic patients with and without dietary management behaviors (DPDM vs. NDPDM), and evaluated the adherence to dietary guidelines in both groups of patients. The data were obtained from the 2002, 2010–2013, and 2015 China National Nutrition Survey. A total of 69,583, 67,177, and 96,631 subjects participated in the 2002, 2010–2013, and 2015 survey rounds, respectively. The dietary intake data were measured using 3-day 24 h dietary recalls and weighed records of household condiments. The China Healthy Diet Index (CHDI) was used to evaluate diet quality. The study included 6229 patients with diabetes, of which 78% had dietary management behaviors. The diabetic patients with dietary management behaviors showed higher percentages of energy from high-quality carbohydrates, animal protein, saturated fatty acids, and unsaturated fatty acids and lower percentages from low-quality carbohydrates and plant protein than NDPDM. The diabetic patients with dietary management behaviors also had lower intakes of cereals and tubers and higher intakes of vegetables than NDPDM. The total CHDI score of DPDM was higher than NDPDM (56.3 ± 12.7 vs. 54.1 ± 12.3). The proportion of DPDM meeting the recommended intake for different food items ranged from 3.3% to 42.8% and from 3.0% to 39.2% in NDPDM. The diabetic patients with dietary management behaviors showed better adherence to dietary guidelines and higher diet quality scores than NDPDM, while the overall adherence was poor in both groups of patients. Our findings suggested that measures are needed to promote and refine dietary management behaviors, which can help to improve disease management in diabetic patients.

## 1. Introduction

Diabetes is one of the most predominant chronic diseases nowadays. In 2021, an estimated 10.5% (537 million) of adults aged 20–79 years were living with diabetes globally, and the number is forecast to grow to 12.2% (783 million) by 2045 [1]. With the aging population and increasing obesity prevalence, diabetes has become an important public health issue in China with a prevalence of 12.8% in 2017 [2]. Dietary management is the cornerstone of diabetes management, which is essential to maintaining glycemic control and reducing the risk of diabetes-related complications [3,4]. Guidelines for diabetes management across different countries have emphasized the importance of conducting dietary management in all patients with diabetes [5,6]. However, adherence to dietary recommendations is a highly challenging task for patients with diabetes, as it usually requires long-term defiance of the patient’s own food cravings and preferences [7,8]. Therefore, it is important to evaluate dietary intake in diabetic patients based on current clinical practice to provide detailed guidance on dietary plans and improve patient management.

Several prior studies have analyzed the dietary intake in diabetic patients and their adherence to dietary guidelines where the dietary pattern and rate of adherence varied greatly in different study populations [9,10,11]. A few studies have analyzed the dietary adherence among diabetic populations in China, where most studies reported low adherence to dietary guidelines [12,13]. However, most of these studies had a small sample size and were not nationally representative. In addition, most previous studies focused on the whole diabetes population without considering whether they had conducted anti-diabetic dietary management behaviors, and these two groups of patients may benefit from different intervention strategies [14]. To the best of our knowledge, few studies have analyzed the implementation of dietary management in Chinese adults with diabetes. 

In this study, we used three rounds of nationally representative data to assess and compare dietary intakes and diet quality between diabetic patients with and without dietary management behaviors (DPDM vs. NDPDM), and evaluated the adherence to dietary guidelines in both groups of patients. This study provided a theoretical basis for promoting and refining dietary management behaviors, which can help to improve disease management in diabetic patients.

## 2. Materials and Methods

### 2.1. Study Population and Sampling

**Figure 1:**
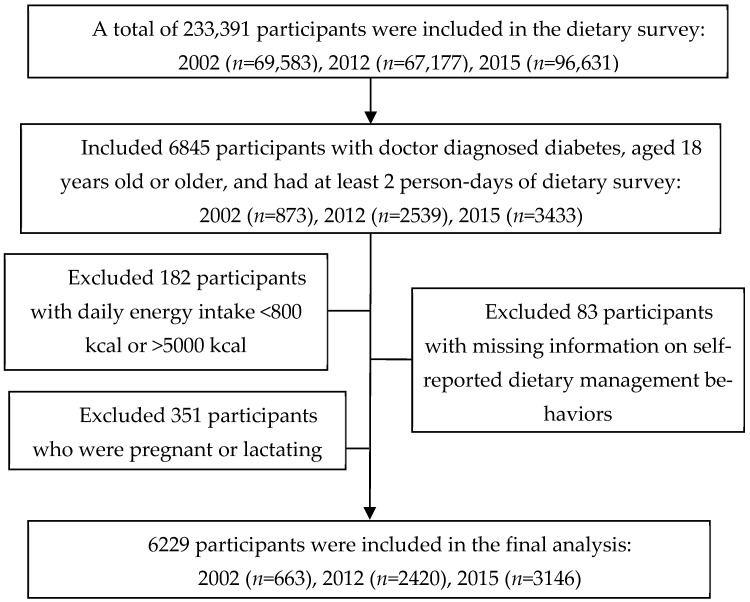
Flow of inclusion into the study.

We analyzed data from the 2002, 2010–2013 (2012), and 2015 China National Nutrition Survey (CNNS), which is one of the most representative national cross-sectional surveys in China. The specific study design has been described elsewhere [15]. Briefly, in the 2002, 2012, and 2015 CNNS, subjects were selected from the resident population of 31 provinces, autonomous regions, and municipalities (except Taiwan, Hong Kong, and Macao) in China. All these studies used multi-stage stratified cluster random sampling. A total of 69,583, 67,177, and 96,631 subjects participated in the dietary survey across the three rounds of CNNS, respectively. The inclusion criteria for this study included: (a) subjects who completed the dietary recall for at least 2 days; (b) aged 18 years or older; (c) those with doctor-diagnosed diabetes. The exclusion criteria were as follows: (a) subjects were pregnant or lactating women; (b) with daily energy intake of less than 800 kcal or greater than 5000 kcal; (c) with missing information on self-reported dietary management behavior. We identified 64,067, 62,960, and 83,538 adults with diabetes that completed at least 2 days of the dietary recall in 2002, 2012, and 2015, respectively, and a total of 6229 patients with diabetes were included in the final analysis (Figure 1). 

The series of CNNS was approved by the ethics committee of the National Institute for Nutrition and Health at the Chinese Center for Disease Control and Prevention (201519-A and 201614). All the subjects signed informed consent before the investigation.

### 2.2. Data Collection and Measurements

Sociodemographic characteristics were collected using questionnaires, including age, gender, marital status, education, occupation, and annual income. The questionnaire also collected data on the diagnosis of diabetes by doctors and whether the subject conducted dietary management behaviors for glycemic control. Height and weight were measured in the morning before breakfast. Fasting plasma glucose and HbA1c were measured by the hexokinase G-6-PDH method and HPLC method, respectively. All measurements were conducted with the Hitachi 7600 automated bio3chemical analyzer and all reagents were produced by Wako Pure Chemical, Ltd. (Richmond, VA, USA). 

### 2.3. Definitions of Diabetic Patients and Dietary Management

In the three CNNS rounds, participants were asked whether they had ever been diagnosed with diabetes, and if they answered yes, they were followed up with questions about what measures they had taken over the past year to control blood sugar. The diabetic patients were defined as participants once diagnosed as having diabetes mellitus. The categorization for dietary management in patients with diabetes was based on the history of dietary management reported in the questionnaire.

### 2.4. Dietary Assessment

Dietary intake was assessed using 24 h dietary recall for three consecutive days (including two weekdays and one weekend) in addition to weighing household cooking oil and condiments. Food intake during the past 24 h was reported by participants for each dietary recall day, and the household cooking oil and condiments were weighed by investigators at the beginning and end of each 24 h. The recall was assisted by an interviewer to ensure accurate information was collected. Nutrient intakes were calculated based on the China Food Composition Tables, which are updated every few years to capture commonly consumed foods and changes in nutrient composition [16,17]. The percentage of participants meeting the recommendations for nutrient intake was evaluated according to the *Guidelines for the Prevention and Treatment of Type 2 Diabetes Milieus in China* (2020 Edition), which recommends 50–65% of total energy intake comes from carbohydrates, 15–20% from proteins, and 20–30% from fat, and daily fiber consumption should be greater than 14 g/1000 kcal [6]. We further subdivided carbohydrates into high-quality carbohydrates and low-quality carbohydrates, proteins into animal proteins and plant proteins, and fats into saturated fatty acids, monounsaturated fatty acids, and polyunsaturated fatty acids, as described in detail in previous articles [18]. Foods were classified into ten major categories according to the 2016 Dietary Guidelines for Chinese Residents, and the percentage of participants meeting the dietary guidelines was evaluated based on the following recommended levels: 250–400 g of cereals and tubers per day; 25–35 g of soybeans and nuts per day; 300–500 g of vegetables per day; 200–350 g of fruits per day; 40–75 g of livestock and poultry meats per day; greater than 300 g of dairy products per day; 40–50 g of eggs per day; 40–75 g of aquatic products per day; 25–30 g of oil per day; and less than 6 g of salt per day [19]. 

We used the China Healthy Diet Index (CHDI) [20] to evaluate the diet quality of the patients. The CHDI was established based on the 2016 *Dietary Guidelines for Chinese Residents* [19,20]. The standards for scoring are presented in Table S1. In this study, we included 13 items, including food variety, refined cereals and tubers, whole grains, dry beans and tubers, total vegetables, dark green and orange vegetables, fruit, dairy, soybeans, meat and egg, fish, shellfish and mollusk, sodium, calories from saturated fatty acids (SFAs), and empty calories. The total score ranged from 0 to 100, with higher scores representing better diet quality. 

### 2.5. Statistical Analysis

We obtained and applied post-stratified population sampling weights derived from the sampling probabilities of the Chinese population aged 18 years or older in 2010 (based on census data) [21]. Means and 95% confidence intervals (CIs) were calculated for weight-adjusted dietary intake. Data were log-transformed before analysis if they were non-normal distribution. The CHDI score was calculated for DPDM and NDPDM in the total population and the stratified population by survey round, age, gender, and area of residence. We compared total energy, the percentages of macronutrients contributing to energy, absolute intake of food groups, and diet quality between DPDM and NDPDM using the Chi-square test for categorical variables, and *t*-test or ANOVA was used for continuous variables. We calculated the adjusted *p*-value using a general linear model to adjust for gender and area and annual income per capita. Two-sided *p* < 0.05 was statistically significant. Statistical analyses were conducted using SPSS 25.0 software (IBM SPSS, Inc., Chicago, IL, USA).

## 3. Results

### 3.1. Participant Characteristics 

A flow diagram of the diabetic patients in the study is presented in Figure 1. A total of 6229 adults with diabetes were included in this study, including 663, 2420, and 3146 patients from the 2002, 2012, and 2015 survey rounds, respectively. The average age was 61.6 years old (range 18.0 to 96.0 years old), and 48.7% of patients were male. The average HbA1c of the included patients was 6.8 ± 1.8% (Table 1). The demographic characteristics were similar across the patients from the three survey rounds (Table S2). In this study, 78.0% of the patients reported having dietary management behaviors. The diabetic patients with dietary management behaviors were more likely to be females, urban residents, and had higher income levels. Body mass index and levels of HbA1c and blood plasma glucose were similar between DPDM and NDPDM. 

### 3.2. Dietary Intake

A comparison of dietary intake between DPDM and NDPDM is shown in Table 2. The total energy was 1756.0 and 1821.1 kcal/day for DPDM and NDPDM, respectively, and no significant difference was observed after adjustment for gender, area, and annual income per capita. After adjustment, compared to NDPDM, DPDM showed a lower estimated percentage of energy from carbohydrates (52.0 vs. 53.5%, *p* = 0.001), specifically a higher estimated percentage of energy from high-quality carbohydrates (5.3 vs. 4.6%, *p* = 0.026) and a lower estimated percentage of energy from low-quality carbohydrates (46.7 vs. 48.8%, *p* < 0.001). Although there was no statistical difference in the estimated percentage of energy from protein between the two groups, DPDM had a higher estimated percentage of energy from animal protein (4.8 vs. 4.3%, *p* = 0.015) and a lower estimated percentage of energy from plant protein (7.4 vs. 7.5%, *p* = 0.030) than NDPDM. The diabetic patients with dietary management behaviors showed a higher estimated percentage of energy from fat (35.4 vs. 33.8%, *p* < 0.001), specifically a higher estimated percentage of energy from saturated fatty acids (8.7 vs. 8.3%, *p* = 0.006), estimated percentage of energy from monounsaturated fatty acids (14.2 vs. 13.5%, *p* = 0.002), and estimated percentage of energy from polyunsaturated fatty acids (11.2 vs. 10.3%, *p* = 0.002) compared to NDPDM. Moreover, DPDM had a lower intake of cereals and tubers (330.2 vs. 363.1 g/day, *p* < 0.001), and a higher intake of vegetables (281.2 vs. 256.4 g/day, *p* < 0.001) and oil (31.9 vs.31.1 g/day, *p* = 0.046) than NDPDM. 

**Figure 2:**
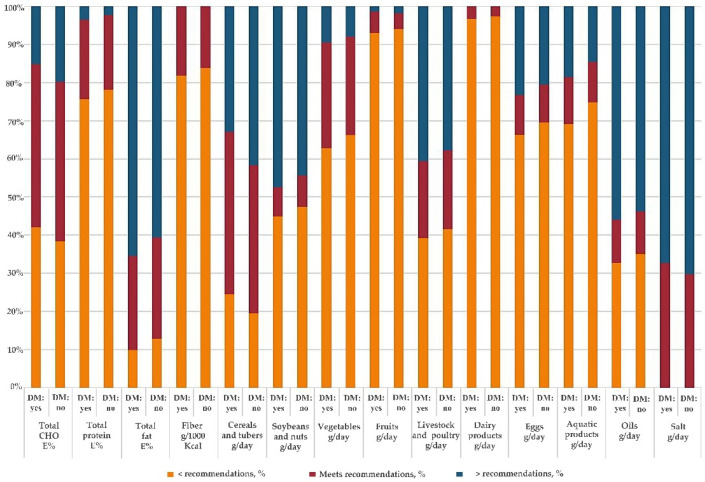
Percentage of adults with diabetes complying with the dietary recommendations in the three rounds of China National Nutrition Surveys. Dietary recommendations in the *Dietary Guidelines for Chinese Residents* (2016): 250–400 g/day cereals and tubers; 25–35 g/day soybeans and nuts; 300–500 g/day vegetables; 200–350 g/day fruits; 40–75 g/day livestock and poultry meats; 40–50 g/day eggs; 40–75 g/day aquatic products; 25–30 g/day oil; >300 g/day dairy products; <6 g/day salt. Dietary recommendations in the *Guidelines for the Prevention and Treatment of Type 2 Diabetes Milieus in China* (2020 Edition): 50–65 E% CHO, 15–20 E% protein, and 20–30 E% fat; >14 g/1000 kcal fiber. Abbreviations: CHO, carbohydrate; E%, percentage of energy; DM: yes, dietary management: yes; DM: no, dietary management: no.

We observed that the majority of patients, both DPDM and NDPDM, did not meet the dietary recommendations (Figure 2). A higher percentage of patients met the dietary recommendations for total carbohydrates (carbohydrates%: 42.6 vs. 41.7%, *p* = 0.005) and fat (fat%: 24.4 vs. 26.9%, *p* = 0.004), while the percentage of patients who met the recommendation for the percentage of energy from protein was around 22% for both groups. Only 18.0% and 15.8% of subjects, respectively, fulfilled the recommendation of fiber intake in DPDM and NDPDM. For the intake of specific food groups, the proportion of DPDM meeting the recommended intake ranged from 3.3% to 42.8% and from 3.0% to 39.2% in NDPDM, DPDM showing an improvement in the intake of vegetables (28.0 vs. 24.7%, *p* = 0.028), cereals and tubers (42.8 vs. 39.2%, *p* < 0.001), and fruits (5.8 vs. 3.7%, *p* = 0.050). In addition, the salt consumption for more than 60% of the subjects was higher than the recommended level in both groups.

### 3.3. Diet Quality Score

The comparison of diet quality scores between DPDM and NDPDM is displayed in Table 3. The total score of DPDM was higher than NDPDM (56.3 ± 12.7 vs. 54.1 ± 12.3, *p* = 0.016). The diabetic patients with dietary management behaviors scored higher than NDPDM on food variety (6.0 ± 2.9 vs. 5.5 ± 3.0, *p* = 0.002) and specific food groups including total vegetables (3.6 ± 1.4 vs. 3.3 ± 1.4, *p* < 0.001), dark green and orange vegetables (2.4 ± 1.9 vs. 2.0 ± 1.8 *p* < 0.001), and meat and egg (3.9 ± 1.6 vs. 3.6 ± 1.8, *p* < 0.001) but scored lower on refined grains (4.6 ± 0.9 vs. 4.7 ± 0.8, *p* = 0.047) and calories from SFAs (8.5 ± 2.9 vs. 8.7 ± 2.7, *p* = 0.022). The difference in total scores between DPDM and NDPDM was observed when stratified by survey year, gender, age, and area of residence, while no differences were observed in the 2015 survey round and the subjects aged below 40 years old subgroups (Table S3). In addition, we noted that subjects who lived in urban areas had higher diet quality scores than those who lived in rural areas, and patients aged ≥65 years had higher scores than patients in other age groups. 

## 4. Discussion

The present study provided a sketch of the implementation of dietary management in patients with diabetes using a series of nationally representative data in China. On the one hand, the overall self-reported rate of having dietary management behaviors was 78%, suggesting that most patients were aware of the need to restrict their dietary intake. Moreover, DPDM showed slight improvements in adherence to dietary guidelines and dietary quality scores compared to NDPDM. On the other hand, we observed large room for improvement in the implementation of dietary management in both groups of diabetic patients where less than half of patients meet the recommended intake level of most food groups, and more scientific guidance on dietary management is required for diabetic patients.

In this study, we found that the majority of patients with diabetes reported having anti-diabetic dietary management behaviors. Previous research showed that the self-reported rate of dietary management behaviors varied across different studies [10,11,22]. For instance, a study in Switzerland found that 51% of diabetic patients reported having an anti-diabetic diet [10], and another study in Italy showed that 56% of patients had self-reported adherence to the anti-diabetic dietary plan [22]. Our findings were consistent with most previous studies in China, where more than half of patients with diabetes reported having dietary management behaviors [23,24]. For instance, a multi-center survey in 2013 showed that 83.8% of diabetic patients reported adherence to dietary management plans [23]. The high rate of self-reported dietary management behaviors was consistent with the treatment guidelines for diabetes where all diabetic patients were recommended to actively engage in dietary management [6]. However, consistent with previous studies [25,26], we observed a relatively low prevalence of the adoption of dietary management among rural patients and patients at lower income levels. Additionally, it is important to note that 69.3% of patients who did not take an anti-diabetic diet were overweight or obese in this study, and these patients were at increased risk for diabetes-related complications [27]. It is therefore still necessary to promote the adoption of self-dietary management in patients with diabetes, especially in rural areas, and with low-income or overweight and obese patients. First, education on self-management for diabetic patients should be enhanced. Such self-management education should be customized and meet the needs of patients, and needs to consider their personal characteristics, health literacy, economic status, and literacy level [28,29]. Second, improving the quality of communication between diabetic patients and healthcare providers, increasing patients’ self-efficacy, and promoting family support are also key to promoting the self-management of diabetic patients [30]. Finally, the Expert Consensus on Diabetes Self-Management Education and Support for Adults with Type 2 Diabetes Mellitus in Primary Healthcare Institutions should be implemented [31], in order to further promote and standardize the development of diabetes self-management education and support (DSMES) in primary care, improve the management and education of patients with type 2 diabetes and their health status, and reduce diabetes-related medical costs. 

We found that DPDM tended to have better adherence to dietary guidelines than NDPDM, though they still require more scientific guidance on dietary management. On the bright side, DPDM had a slightly lower total energy intake compared to NDPDM. The estimated energy from carbohydrates was decreased and the estimated energy from protein and fat was increased in DPDM compared to NDPDM. More specifically, DPDM showed higher energy intake from high-quality carbohydrates, monounsaturated fatty acids, and polyunsaturated fatty acids, and lower energy intake from low-quality carbohydrates than NDPDM. In addition, DPDM had higher diet quality compared to NDPDM, and they scored higher than NDPDM on food variety and specific food groups including total vegetables, dark green and orange vegetables, and meat and eggs but scored lower on refined grains. These results were consistent with the dietary guidelines for diabetic patients, which recommended reducing overall carbohydrate intake and increasing the proportion of unsaturated fats and high-quality carbohydrate intake, as well as the intake of food high in fiber, such as nonstarchy vegetables and whole grains [5,6]. Similarly, a study conducted in Switzerland found that participants who reported an anti-diabetic diet had a higher consumption of vegetables compared to those not on a diet, while they did not observe significant differences in all other food groups and nutrients [10]. However, it is worth noting that the difference between DPDM and NDPDM in some perspectives did not follow the dietary guidelines. Despite various studies suggesting that a plant protein diet instead of an animal protein diet was beneficial to glycemic control [32], we observed that DPDM showed higher energy intake from animal protein and saturated fatty acids, and lower CHDI scores on calories from SFAs compared to NDPDM, which may be related to the higher intake of livestock and poultry meats in these subjects. These findings were consistent with several previous observational studies, which also reported a higher level of saturated fat intake than the recommendations in patients with diabetes [33,34,35]. In addition, we observed that the proportion of patients with lower than recommended carbohydrate intake was higher in DPDM compared to NDPDM. This finding suggested that some patients were too conscious on the quantity of carbohydrate intake, which may negatively influence dietary management [36]. These mismatches to dietary guidelines observed in this study suggested that more scientific guidance on dietary management was required for patients with diabetes, especially on detailed instructions for achieving a balanced diet.

Despite the general improvement in diet among DPDM, this study showed that the implementation of dietary management against diabetes was far below satisfactory in China. The majority of patients failed to meet the dietary recommendations for the intake of carbohydrates, protein, fat, and fiber, and even in DPDM only 3.3%, 5.8%, 27.9% and 32.4% of patients meet the recommended intake of dairy, fruits, vegetables, and salt, respectively. Low adherence to dietary recommendations has been reported by several studies, despite the variation of dietary guidelines between nations [10,11,37]. A systematic review in 2017 found that the majority of participants consumed less than the recommended servings of fruit, vegetables, grains, and dairy based on 11 cross-sectional studies in eight countries [38]. Moreover, our findings showed a significantly lower dietary quality than the recommended level in both patient groups, which was also true in Mexican adults with diabetes [39]. Moreover, studies in China also reported that the overall dietary quality remained poor in the population, where most people had an inadequate intake of milk and dairy products, nuts, fruits, other cereals and miscellaneous beans, and seafood compared with the recommended intake levels [40,41]. The implementation of dietary management in diabetic patients has long been considered a difficult task [37,42]. Previous studies reported that dietitian involvement or carbohydrate counting was not common in China, and patients usually paid more attention to medical treatments instead of lifestyle interventions [43,44]. The implementation of dietary management in China is challenging considering the large diabetic population, and a great proportion of patients live in rural areas or have low education levels [2]. Over the years, the ministry of health in China has tried to improve education on self-management for patients with chronic diseases through community healthcare centers [45], and we also found that the majority of participants in our study reported having dietary management behaviors. However, our findings also suggested that current education was not enough for patients to change their lifestyles, and many DPDM have an unbalanced intake of nutrients and foods. It is therefore important for the healthcare system to conduct education and management programs for patients with diabetes and truly help patients improve their dietary habits.

In this study, we used three rounds of nationally representative data to assess and compare the dietary intakes and diet quality between DPDM and NDPDM, and evaluated the adherence to dietary guidelines in both groups of patients. To the best of our knowledge, this is the first study in China that compared the composition of diets between DPDM and NDPDM using an unselected, population-based study. In the meantime, we acknowledge that this study had certain limitations. First, dietary intake was collected using a three-day dietary recall, while patients’ dietary habits may change over time along the disease process. Thus, it is necessary to conduct longitudinal studies in the future to capture the difference in dietary intake over time. Second, this was a cross-sectional study; thus, we were not able to make causal inferences regarding dietary behaviors and dietary intake in diabetic patients. Third, we were not able to distinguish between type 1 and type 2 diabetes in this study. However, as type 2 diabetes accounts for more than 90% of all diabetes cases in adults [46], most of the cases in this study were likely type 2 diabetes. Fourth, we cannot rule out some confounding factors that were not included in this analysis, such as antidiabetic medications. 

In conclusion, according to our data, 78% of Chinese adults with diabetes adopted dietary management behaviors, but the prevalence was relatively lower in rural, low-income, obese and overweight patients. The diabetic patients with dietary management behaviors showed better adherence to dietary guidelines and higher diet quality scores than NDPDM, while there was large room for improvement in both groups. Our findings suggested that more effective strategies and measures are needed to purposefully promote and refine the implementation of dietary management in patients with diabetes. 

## Figures and Tables

**Table 1:** Characteristics of adults with diabetes in the three rounds of China National Nutrition Surveys.

Variable	Overall *n* = 6229	Dietary Management: Yes *n* = 4857	Dietary Management: No *n* = 1372	*p*
Gender				
Male	2801 (48.7)	2118 (47.1)	683 (54.3)	<0.001
Female	3428 (51.3)	2739 (52.9)	689 (45.7)	
Age group (years)				
18–39	150 (6.3)	113 (6.1)	37 (6.9)	0.250
40–64	3681 (61.6)	2844 (61.1)	837 (63.4)	
≥65	2398 (32.1)	1900 (32.8)	498 (29.7)	
Area				
Urban	4133 (69.2)	3332 (71.2)	801 (62.3)	<0.001
Rural	2096 (30.8)	1525 (28.8)	571 (37.7)	
Annual income per capita				
Very low	1089 (17.4)	788 (16.2)	301 (21.6)	0.002
Low	1150 (18.4)	867 (18.0)	283 (19.9)	
Middle	1257 (20.9)	982 (21.0)	275 (20.6)	
High	1161 (20.1)	936 (20.5)	225 (18.7)	
Very high	1398 (23.2)	1145 (24.3)	253 (19.2)	
Education level				
Under primary	912 (12.7)	693 (12.4)	219 (14.0)	0.646
Primary school	1329 (21.0)	1024 (20.7)	305 (21.6)	
Junior high school	1574 (26.0)	1231 (26.3)	343 (24.9)	
Senior high school	1463 (23.9)	1148 (22.9)	315 (24.1)	
Junior college and above	951 (16.4)	761 (16.7)	190 (15.4)	
Marital status				
Single	36 (1.3)	25 (1.1)	11 (2.2)	0.073
Married/Cohabiting	5571 (88.8)	4352 (89.2)	1219 (87.3)	
Widowed/Divorced/Separated	621 (9.9)	479 (9.7)	142 (10.5)	
Body mass index (kg/m^2^)				
<18.5	95 (1.7)	74 (1.8)	21 (1.6)	0.217
18.5–23.9	1925 (32.3)	1537 (33.1)	388 (29.1)	
24–27.9	2607 (42.9)	2018 (42.2)	589 (45.4)	
≥28	1397 (23.1)	1073 (22.9)	324 (23.9)	
HbA1c (%)	6.8 ± 1.8	6.8 ± 1.8	6.8 ± 1.8	0.411
Fasting Plasma Glucose (mmol/L)	8.3 ± 3.2	8.2 ± 3.2	8.4 ± 3.5	0.134

Data are *n* (weighted%) or weighted mean ± SD. Number missing: Body mass index (*n* = 205), Marital status (*n* = 1), Annual income per capita (*n* = 174), HbA1c (*n* = 2431). Abbreviations: HbA1c, glycated hemoglobin. Comparison of characteristics between diabetic patients with and without dietary management was conducted using the *t*-test for continuous variables and the Chi-square test for categorical variables.

**Table 2:** Mean intake of dietary-related items among Chinese adults with diabetes in the three rounds of China National Nutrition Surveys.

Variable	Guideline Targets ^1^	Weighted Mean (95% CI)		*p*	Adjusted *p*-Value ^2^
		Dietary Management: Yes	Dietary Management: No		
Energy and nutrients ^3^					
Energy intake, kcal/day		1756.0	1821.1	0.007	0.186
		(1734.2–1777.9)	(1778.8–1864.2)		
Total carbohydrates, E%	50–65	52.0	53.5	0.004	0.001
		(51.5–52.5)	(52.6–54.4)		
High-quality carbohydrates, E%		5.3	4.6	<0.001	0.026
		(5.1–5.5)	(4.3–5.0)		
Low-quality carbohydrates, E%		46.7	48.8	<0.001	<0.001
		(46.2–47.1)	(47.9–49.8)		
Total protein, E%	15–20	13.1	12.8	0.007	0.178
		(13.0–13.3)	(12.5–13.0)		
Animal protein, E%		4.8	4.3	<0.001	0.015
		(4.7–4.9)	(4.0–4.5)		
Plant protein, E%		7.4	7.5	0.176	0.030
		(7.3–7.5)	(7.3–7.7)		
Total fat, E%	20–30	35.4	33.8	0.001	<0.001
		(35.0–35.9)	(32.9–34.7)		
Saturated fatty acids, E%		8.7	8.3	0.006	0.006
		(8.5–8.8)	(8.0–8.5)		
Monounsaturated fatty acids, E%		14.2	13.5	0.007	0.002
		(14.0–14.5)	(13.1–14.0)		
Polyunsaturated fatty acids, E%		11.2	10.3	<0.001	0.002
		(10.9–11.4)	(9.9–10.7)		
Fiber, g/1000 kcal	>14	8.6	8.4	0.269	0.618
		(8.4–8.7)	(8.0–8.7)		
Food groups					
Cereals and tubers, g/day	250–400	330.2	363.1	<0.001	<0.001
		(324.5–324.5)	(351.1–375.6)		
Soybeans and nuts, g/day	25–35	59.0	50.4	0.002	0.060
		(56.0–62.0)	(45.8–54.9)		
Vegetables, g/day	300–500	281.2	256.4	<0.001	<0.001
		(274.8–287.7)	(245.2–267.6)		
Fruits, g/day	200–350	105.1	117.7	0.345	0.506
		(84.5–130.8)	(63.3–218.9)		
Livestock and poultry meats, g/day	40–75	83.5	79.4	0.231	0.076
		(80.3–86.7)	(73.0–85.8)		
Dairy products, g/day	>300	141.9	134.6	0.452	0.815
		(133.6–150.6)	(119.1–152.1)		
Eggs, g/day	40–50	48.1	49.3	0.626	0.411
		(41.0–56.3)	(35.3–68.8)		
Aquatic products, g/day	40–75	39.1	35.0	0.137	0.588
		(36.5–41.7)	(30.4–39.7)		
Oil, g/day	25–30	31.9	31.1	0.414	0.046
		(31.0–32.8)	(29.5–32.8)		
Salt, g/day	<6	7.6	8.1	0.079	0.187
		(7.5–7.9)	(7.7–8.5)		

Data were adjusted for China National Nutrition Survey weights to be nationally representative. Comparison of dietary intake between diabetic patients with and without dietary control was conducted using the *t*-test. ^1^ Level of recommendations was based on the *Dietary Guidelines for Chinese Residents* (2016) and *Guidelines for the Prevention and Treatment of Type 2 Diabetes Milieus in China* (2020 Edition). ^2^ This was adjusted for gender, area, and annual income per capita using general linear models. ^3^ High-quality carbohydrates were defined as carbohydrates from whole grains, fruits, legumes, and non-starchy vegetables. Low-quality carbohydrates were defined as carbohydrates from refined grains, added sugars, tubers, other starchy vegetables, and other sources. Animal protein was defined as protein from aquatic products, livestock and poultry meats, dairy products, eggs, and other sources. Plant protein was defined as protein from whole grains, refined grains, legumes, nuts, and other sources.

**Table 3:** CHDI components and criteria for scoring and the score of adults with diabetes in the three rounds of China National Nutrition Surveys.

CHDI Component	Score Range	Standard for Maximum Score	Standard for Minimum Score of Zero	Weighted Mean ± SD		*p*	Adjusted *p*-Value ^1^
				Dietary Management: Yes	Dietary Management: No		
Food variety	0–10	≥12 kind	≤5 kind	6.0 ± 2.9	5.5 ± 3.0	<0.001	0.002
Refined grains	0–5	≥100 g/1000 kcal	0	4.6 ± 0.9	4.7 ± 0.8	0.033	0.047
Whole grain, dry bean, and tuber	0–5	≥40 g/1000 kcal	0	2.7 ± 2.1	2.7 ± 2.1	0.983	0.738
Total vegetables	0–5	≥180 g/1000 kcal	0	3.6 ± 1.4	3.3 ± 1.4	<0.001	<0.001
Dark green and orange vegetables	0–5	≥90 g/1000 kcal	0	2.4 ± 1.9	2.0 ± 1.8	<0.001	<0.001
Fruit	0–10	≥110 g/1000 kcal	0	1.8 ± 1.4	1.8 ± 1.4	0.801	0.114
Dairy	0–10	≥100 g/1000 kcal	0	6.4 ± 1.9	6.3 ± 1.8	0.670	0.917
Soybean	0–10	≥10 g/1000 kcal	0	5.7 ± 4.8	5.6 ± 4.8	0.335	0.192
Meat and egg	0–5	≥50 g/1000 kcal	0	3.9 ± 1.6	3.6 ± 1.8	<0.001	<0.001
Fish, shellfish and mollusk	0–5	≥30 g/1000 kcal	0	3.6 ± 1.9	3.5 ± 1.9	0.872	0.171
Calories from SFAs	0–10	<10%	≥15%	8.5 ± 2.9	8.7 ± 2.7	0.091	0.022
Sodium	0–10	≤1 g/1000 kcal	≥4 g/1000 kcal	4.5 ± 3.3	4.5 ± 3.3	0.958	0.690
Empty calories	0–10	≤20%	≥40%	8.5 ± 2.9	8.7 ± 2.8	0.279	0.123
Total	0–100			56.3 ± 12.7	54.1 ± 12.3	<0.001	0.016

Data were adjusted for China National Nutrition Survey weights to be nationally representative. Abbreviations: CHDI, China Healthy Diet Index; SFAs, saturated fatty acids; SD, standard deviation. Comparison of scores between diabetic patients with and without dietary management was conducted using the *t*-test. ^1^ Scores were adjusted for gender, area, and annual income per capita using general linear models.

## Data Availability

The data are not allowed to be disclosed according to the National Institute for Nutrition and Health and the Chinese Center for Disease Control and Prevention.

## Supplementary Materials

The following supporting information can be downloaded at: https://www.mdpi.com/article/10.3390/nu14235178/s1, Table S1: CHDI components and criteria for scoring; Table S2: Characteristics of adults with diabetes across the three rounds of China National Nutrition Surveys; Table S3: CHDI score of adults with diabetes in the three rounds of China National Nutrition Surveys. Reference [20] is cited in the supplementary materials.
