# Supplementary material for: Self-Reported Dietary Management Behaviors and Dietary Intake among Chinese Adults with Diabetes: A Population-Based Study
Source: Nutrients. 2022 Dec 5;14(23):5178. doi: 10.3390/nu14235178 (PMC9740534; doi:10.3390/nu14235178)
Supplement: Supplementary file 1 [file nutrients-14-05178-s001.zip › nutrients-2006969-supplementary.pdf]

Supplementary material

Self-reported dietary management behaviors and dietary intake among Chinese adults with diabetes: a population-based study

Yixu Liu [1,†], Dongmei Yu [2,†], Jiayou Luo [1], Shuya Cai [2], Ping Ye [3], Zhenzhen Yao [1], Miyang Luo [1,*] and Liyun Zhao [2,*]

[1] Xiangya School of Public Health, Central South University, Changsha 410008, China
[2] Key Laboratory of Trace Element Nutrition of National Health Commission, Chinese Center for Disease Control and Prevention, National Institute for Nutrition and Health, Beijing 100050, China
[3] Yuxi Center for Disease Control and Prevention, Yuxi 653100, China
[*] Correspondence: miyangluo@csu.edu.cn; zhaoly@ninh.chinacdc.cn

[†] These authors contributed equally to this work.

Contents

Table S1. CHDI components and criteria for scoring.
Table S2. Characteristics of adults with diabetes across the three rounds of China National Nutrition Surveys.
Table S3. CHDI score of adults with diabetes in the three rounds of China National Nutrition Surveys.

Table S1. CHDI components and criteria for scoring [1].

CHDI Component	Score Range	Standard for Maximum Score	Standard for Minimum Score of Zero
Food variety	0–10	≥12 kind	≤5 kind
Refined grains	0–5	≥100 g/1000kcal	0
Whole grain, dry bean, and tuber	0–5	≥40 g/1000kcal	0
Total vegetables	0–5	≥180 g/1000kcal	0
Dark green and orange vegetables	0–5	≥90 g/1000kcal	0
Fruit	0–10	≥110 g/1000kcal	0
Dairy	0–10	≥100 g/1000kcal	0
Soybean	0–10	≥10 g/1000kcal	0
Meat and egg	0–5	≥50 g/1000kcal	0
Fish, shellfish and mollusk	0–5	≥30 g/1000kcal	0
Calories from SFAs	0–10	<10%	≥15%
Sodium	0–10	≤1 g/1000kcal	≥4 g/1000kcal
Empty calories	0–10	≤20%	≥40%

Abbreviations: CHDI, China Healthy Diet Index; SFAs, saturated fatty acids.

Table S2. Characteristics of adults with diabetes across the three rounds of China National Nutrition Surveys.

Variable	2002 (*n*=663)	2012 (*n*=2420)	2015 (*n*=3146)
Gender			
Male	297 (48.5)	1093 (49.7)	1411 (47.8)
Female	366 (51.5)	1327 (50.3)	1735 (52.2)
Age group (years)			
18-39	33 (6.3)	66 (7.2)	51 (5.4)
40-64	424 (66.6)	1407 (60.7)	1850 (60.2)
≥65	206 (27.1)	947 (32.1)	1245 (34.4)
Area			
Urban	467 (77.6)	1775 (68.5)	1891 (65.9)
Rural	196 (22.4)	645 (31.5)	1255 (34.1)
Annual income per capita			
Very low	60 (9.4)	429 (20.2)	600 (18.4)
Low	103 (14.7)	399 (18.8)	648 (19.8)
Middle	169 (25.9)	473 (20.0)	615 (19.6)
High	189 (30.2)	328 (13.5)	644 (21.5)
Very high	122 (19.8)	637 (27.5)	639 (20.7)
Education level			
Under primary	68 (9.3)	324 (12.6)	520 (14.5)
Primary school	210 (28.1)	699 (26.9)	420 (11.6)
Junior high school	197 (30.9)	749 (30.9)	628 (18.7)
Senior high school	131 (21.7)	435 (19.6)	897 (29.4)
Junior college and above	57 (10.0)	213 (10.0)	681 (25.8)
Marital status			
Single	9 (2.2)	12 (1.1)	15 (1.1)
Married/Cohabiting	584 (87.9)	2087 (86.7)	2900 (91.4)
Widowed/Divorced/Separated	69 (9.9)	321 (12.2)	231 (7.5)
Body mass index (kg/m²)			
<18.5	14 (1.9)	41 (2.2)	40 (1.3)
18.5-23.9	225 (34.6)	767 (34.3)	933 (29.1)
24-27.9	273 (41.9)	970 (43.3)	1364 (43.1)
≥28	148 (21.6)	440 (20.2)	809 (26.5)
Dietary management behavior			
Yes	536 (80.9)	1915 (78.0)	2406 (76.3)
No	127 (19.1)	505 (22.0)	740 (23.7)

Data are *n* (weighted%). Number missing: Body mass index (*n*=205), Marital status (*n*=1), Annual income per capita (*n*=174).

Table S3. CHDI score of adults with diabetes in the three rounds of China National Nutrition Surveys.

Variable	weighted mean ± SD		*p*
	Dietary management: yes	Dietary management: no	
All	56.3 ± 12.7	54.1 ± 12.3	<0.001
Year			
2002	57.5 ± 12.1	53.9 ± 11.0	0.001
2012	56.1 ± 12.5	53.7 ± 12.0	0.004
2015	56.0 ± 13.1	54.6 ± 13.1	0.104
Gender			
Male	57.0 ± 12.3 *	54.8 ± 12.1	0.001
Female	55.7 ± 13.0 *	53.4 ± 12.6	0.003
Age group, years			
18-39	55.9 ± 11.5 **	53.0 ± 13.0 *	0.128
40-64	55.5 ± 12.5 **	53.4 ± 11.6 *	0.001
≥65	58.0 ± 13.1 **	55.9 ± 13.6 *	0.038
Area			
Urban	58.5 ± 12.8 **	57.0 ± 12.1 **	0.023
Rural	51.0 ± 10.6 **	49.4 ± 11.2 **	0.034

Data were adjusted for China National Nutrition Surveys weights to be nationally representative. Comparison of CHDI scores within each subgroup was conducted using the *t*-test or ANOVA, * $p<0.05$, ** $p<0.001$. Comparison of the score between diabetic patients with and without dietary management was conducted using the *t*-test. Abbreviations: CHDI, China Healthy Diet Index; SD, standard deviation.

References

1. He, Y.; Fang, Y.; Yang, X. Establishment and application of China healthy diet inde x. *Acta Nutrimenta Sinica* **2017**, *39*, 436-441. https://doi.org/10.13325/j.cnki.acta.nutr.sin.2017.05.011.
